# Evaluating Google Trends as a proxy for symptom incidence: insights from the winter COVID-19 infection study in England 2023/24

**DOI:** 10.1017/S0950268825100794

**Published:** 2025-11-28

**Authors:** Phoebe Asplin, Martyn Fyles, Jack Kennedy, Thomas Ward, Jonathon Mellor

**Affiliations:** 1 UK Health Security Agency, UK; 2 University of Warwick, UK

**Keywords:** Community surveillance, symptom, internet search

## Abstract

Google Trends is used in research and surveillance as a proxy for community infection incidence. Signals are difficult to validate, as most surveillance biases towards severe outcomes and certain demographics.

Using Winter COVID-19 Infection Study (WCIS) data in England, symptom prevalence is estimated via generalized additive model with multilevel-regression and poststratification. Symptom duration was estimated using interval censored time delay modelling, converting prevalence to incidence. Google Trends and WCIS incidence and growth rates were compared using cross-correlation.

Google Trends and WCIS agreement varied by symptom and age group. The national maximum growth rate cross-correlation for sore throat was 0.81, with 90% prediction intervals of [0.69, 0.90]. Google Trends growth rates generally lagged the WCIS growth rates across symptoms (cough: −5.0 days [−8.0, 0.0], fever: −3.0 days [−6.0, 1.0], loss of smell: −9.0 days [−13, −3.0], shortness of breath: −12 days [−16, −5.0], and sore throat: −4.0 days [−5.0, −2.0]).

This work shows Google Trends and community symptom incidence can align, although substantial variation between symptoms and age groups exists, underscoring utility in predicting other surveillance indicators.

## Introduction

Infections in community settings pose a challenge for surveillance due to the high resource requirements to observe them. Traditional epidemiological case surveillance relies on symptomatic presentation in to healthcare providers, biasing trends away from the general population towards individuals more likely to present severe outcomes. As a result, new infections in the community are challenging to estimate from traditional surveillance data. This problem has been tackled in numerous ways, such as community infection studies [1], and the analysis of proxy signals [2, 3].

Large community infection studies mitigate this severity bias by sampling individuals independent of symptom status [4]. This reduction in bias means the positivity and prevalence of an infection or symptom can be ascertained via a survey sent to participants. With further data on the duration of infection or symptoms, the incidence of new cases can be calculated, a key epidemic quantity for understanding disease burden and downstream metrics [5].

The Winter COVID-19 Infection Study (WCIS) is an example of such a community incidence study, run jointly by the UK Health Security Agency and the Office for National Statistics [5]. The study was performed between 13 November 2023 and 7 March 2024 across England and involved 111,752 participants. Participants were asked to complete a SARS-CoV-2 lateral flow test and list the symptoms they had in the last 7 days, in addition to their symptom onset date. Thus, from this data, an estimate for the community incidence of specific symptoms can be obtained.

However, these community infection studies are rare and costly in practice. This motivates the use of proxy signals, which are abundant, but also introduce their own biases. Much work has been done on both community infection studies and the analysis of proxy signals during the SARS-CoV-2 pandemic, leading to large amounts of data, but room for further understanding.

The use of proxy signals to monitor community incidence in England dates back to the late 1990s, with telephone health lines being used for surveillance of ‘cold or flu’ symptoms [6, 7]. The number of calls from patients presenting these symptoms increased at times when influenza was known to be circulating. English syndromic surveillance was further expanded during the 2009 influenza pandemic to include GP consultation surveillance [8]. In more recent times, this has been further expanded to include the NHS 111 online syndromic surveillance system. However, this system is dependent on the NHS and can require a high volume of logistical work to set up and maintain these systems. Furthermore, these systems are set up only in the UK and cannot contribute towards a unified international syndromic surveillance system. This motivates the use of Google Trends as a proxy for community symptom incidence, due to its global availability and the minimal costs associated with its use.

Google Flu Trends was launched in November 2008 as a tool for influenza surveillance in the United States [9]. Whilst the tool had limited success – and was discontinued in 2015 [10] – it paved the way for more advanced digital surveillance systems. One of these systems is the Google COVID-19 Search Trends symptoms dataset (henceforth referred to as ‘Google Trends’), which was started in 2020 in response to the COVID-19 pandemic [11].

Google Trends and its precursors have been used widely as a proxy for infection presentation. Conceptually, if more individuals are searching for an infection-related keyword, one might expect a higher prevalence of infections in the community. Numerous works have descriptively compared Trends to severity-biased indicators [12–16]. Limited research has sought to understand the relationship between Google Trends and community incidence patterns, which underpins the disease dynamics of most predictive modelling.

In this work, we produce estimates for the incidence of new symptoms in the community using WCIS data [5], by converting prevalence and symptom duration data into incidence rates with a representative longitudinal cohort. With this ground-truth incidence estimate stratified by age, we compare the time series of Google Trends in England to understand how well the internet search data serves as a proxy for community symptom incidence.

## Results

We analysed Google Trends data and compared it with age-stratified symptom incidence estimates from WCIS data. First, we summarize the estimated symptom durations used to convert WCIS prevalence data into incidence rates. We then compare the temporal patterns and magnitudes of Google Trends search volumes with the WCIS-derived symptom incidence. To assess whether the two datasets exhibit similar directional trends, we compare the growth rates of their respective incidence estimates. Finally, we assess the relative timing of the two datasets using cross-correlation analysis to determine whether the growth rate of Google Trends incidence tends to lead or lag behind changes in the WCIS incidence growth rate.

The estimates for the symptom durations from WCIS are shown in Table 1. The mean and probability mass distributions by symptom and age group are shown in Supplementary Figures 1 and 2. The estimates for WCIS symptom prevalence are shown in Supplementary Figure 3.

**Table 1.** Mean and 90% credible interval of the symptom duration estimate, in days, stratified by age group

Symptom	3 to 17 years	18 to 34 years	35 to 44 years	45 to 54 years	55 to 64 years	65 to 74 years	75 years and over
Cough	12.4 [12.1, 12.8]	12.0 [11.6, 12.4]	12.7 [12.5, 13.0]	13.7 [13.5, 13.9]	14.9 [14.7, 15.1]	16.4 [16.2, 16.6]	17.5 [17.1, 17.8]
Fever	10.3 [9.75, 10.9]	10.0 [9.56, 10.5]	9.94 [9.62, 10.3]	10.7 [10.4, 11.0]	10.8 [10.6, 11.1]	11.3 [11.0, 11.7]	12.0 [11.5, 12.6]
Sore throat	10.2 [9.86, 10.6]	9.40 [9.11, 9.67]	9.62 [9.44, 9.80]	9.95 [9.79, 10.1]	10.7 [10.6, 10.9]	11.7 [11.5, 11.9]	12.9 [12.6, 13.2]
Shortness of breath	13.3 [12.2, 14.6]	12.8 [12.1, 13.6]	14.0 [13.4, 14.6]	15.0 [14.5, 15.4]	17.3 [16.9, 17.8]	20.4 [19.8, 21.1]	23.7 [22.7, 24.9]
Loss of smell	12.4 [10.9, 14.3]	11.5 [10.7, 12.4]	11.7 [11.2, 12.2]	13.0 [12.5, 13.6]	14.4 [13.9, 14.9]	16.1 [15.4, 16.7]	18.0 [16.8, 19.3]

**Figure 1.**
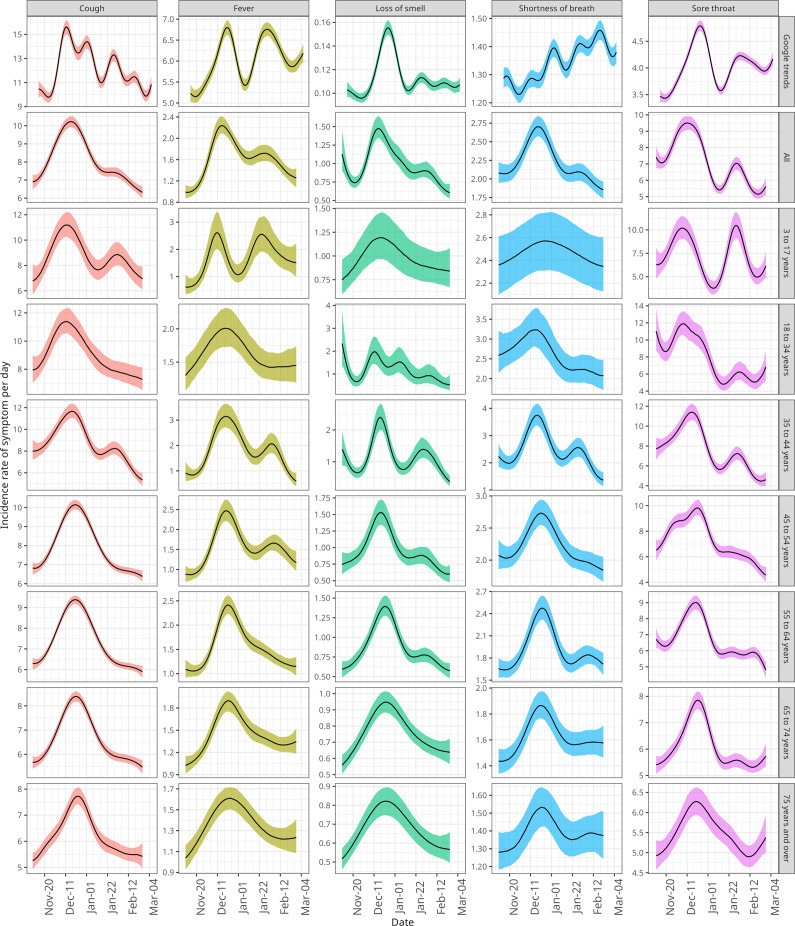
Daily symptom incidence over time for Google Trends and WCIS symptoms. Median estimates and 90% prediction intervals are shown. WCIS estimates are stratified by age group and across all ages (national) and presented as a per capita rate per 1,000 population. Google Trends is a relative search volume.

We first compare incidence estimates from Google Trends with those from WCIS (nationally and by age group, Figure 1). For most symptoms, the trajectory of incidence was similar between Google Trends and the national WCIS estimate. However, we did find that peaks in Google incidence generally lagged the national WCIS estimate.

For cough, loss of smell, and sore throat, we consistently observed a large initial peak in late December, followed by a smaller second peak. For fever, we saw a similar two-peak pattern in both incidence estimates, although the second peak was proportionally larger in the Google estimate, reaching a similar level to the initial peak. In contrast, we found large variations in the incidence of shortness of breath between the Google and national WCIS estimates. Whilst for WCIS we observed a similar initial peak to that seen for the other symptoms, the Google incidence instead generally increased throughout the whole of the study period.

One notable outcome of our WCIS incidence estimates was the variation between age groups. For example, regarding cough and fever, the maximum incidence during the second peak was more similar to the first peak for the 3 to 17 and 35 to 44 years age groups compared to the others. The relative heights of the two peaks in these two age groups aligned more closely with the Google incidence than the national WCIS incidence. On the other hand, we saw no second peak in incidence for the three oldest age groups across any of the symptoms considered. Since poststratification was performed for WCIS but not for Google Trends, it is likely that the age distribution of individuals who use Google (which is unavailable) is a contributing factor to the differences between Google Trends and national WCIS incidence.

**Figure 2.**
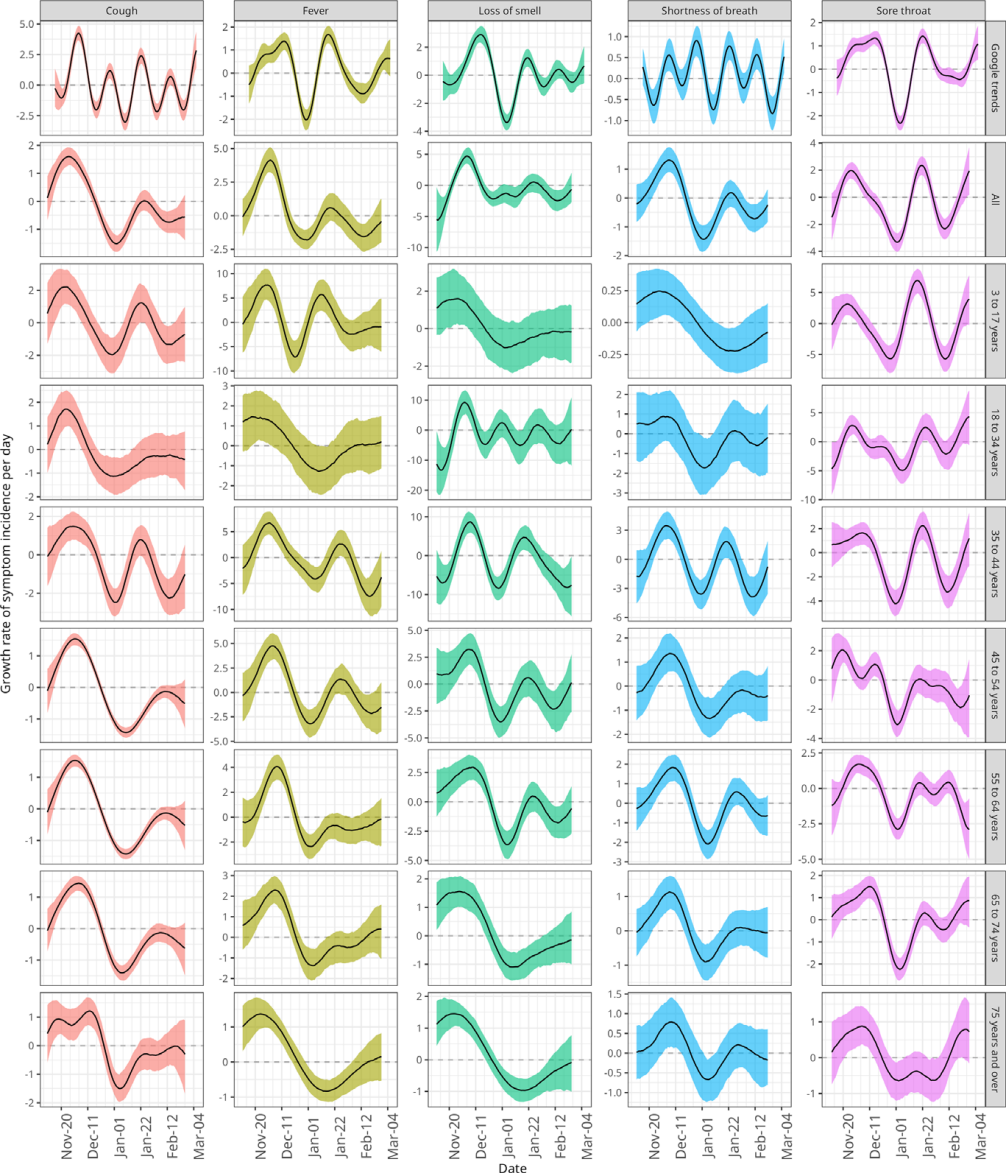
Daily symptom incidence growth rate over time for Google Trends and WCIS symptoms. Median estimates and 90% prediction intervals are shown. WCIS estimates are stratified by age group and across all ages (national). Both Google and WCIS growth rate estimates are presented as a daily percentage change.

We then compared the Google Trends growth rate with that of WCIS (Figure 2). Similarly to our previous findings, we found Google Trends growth rates were qualitatively similar to the national WCIS growth rates, consistently seeing two peaks which occurred at similar times. The growth rates being similar between the two datasets indicate the direction of incidence aligns, despite differences in magnitudes. We also found similarities in the growth rates for shortness of breath between the two datasets, despite the incidence being quite different. In this case, for both Google and WCIS, there were two periods during which the growth rate increased, separated by three time points at which the growth rate was negative. Although the timing of these minimum points did not align exactly (again, the Google Trends dynamics are lagged), the time between these points was consistent between the datasets.

**Figure 3.**
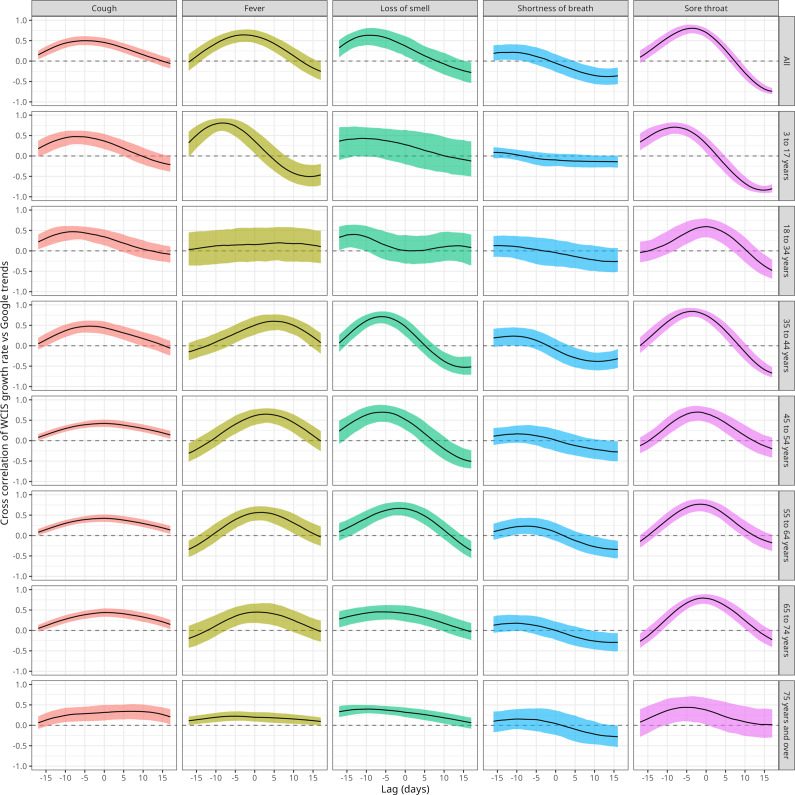
Cross-correlation at lags −17 to 17 between the Google Trends symptom growth rate and WCIS growth rates by age group stratification, with median estimate and 90% prediction intervals.

We then examined the cross-correlation between the WCIS and Google Trends growth rates (Figure 3). Summary statistics are also presented in Tables 2 and 3, with the median estimate and 90% prediction interval given for the maximum cross-correlation and the lag at which that maximum was reached. Across symptoms, the median growth rate cross-correlation for national WCIS was at a maximum for a negative lag (cough: −5.0 days [−8.0, 0.0], fever: −3.0 days [−6.0, 1.0], loss of smell: −9.0 days [−13, −3.0], shortness of breath: −12 [−16, −5.0], and sore throat: −4.0 days [−5.0, −2.0]) meaning that the Google signal most likely lags WCIS by at least 3 days. Across age groups, the median lag with the maximum growth rate cross-correlation was consistently negative or zero, apart from fever, for which the median lag was positive for ages 18 to 74. These results quantify our findings from Figures 1 and 2, where we saw that the peaks in the Google signal occurred later than the peaks in WCIS incidence.

**Table 2.** Median and 90% prediction interval of the lag, in days, with the maximum cross-correlation between the growth rate of Google Trends and WCIS age stratifications

Lag with maximum growth rate cross-correlation								
Symptom	National	3 to 17 years	18 to 34 years	35 to 44 years	45 to 54 years	55 to 64 years	65 to 74 years	75 years and over
Cough	−5.0 [−8.0, 0.0]	−7.0 [−12, 0.0]	−8.0 [−12, 1.0]	−4.0 [−8.0, 3.0]	0.0 [−4.0, 2.0]	0.0 [−4.0, 2.0]	0.0 [−4.0, 6.0]	4.5 [−10, 15]
Fever	−3.0 [−6.0, 1.0]	−8.0 [−12, −5.0]	3.0 [−17, 17]	5.0 [0.0, 9.0]	3.0 [−2.0, 7.0]	1.0 [−2.0, 6.0]	1.0 [−6.0, 8.0]	−4.0 [−10, 13.]
Loss of smell	−9.0 [−13, −3.0]	−9.0 [−17, 12]	−12. [−17, 12]	−6.0 [−9.0, −4.0]	−6.0 [−11, −2.0]	−2.0 [−6.0, 2.0]	−5.0 [−14, 4.0]	−10. [−15, 0]
Shortness of breath	−12. [−16, −5.0]	−16. [−16, 5.0]	−12. [−16, 8.0]	−11. [−16, −5.0]	−9.0 [−16, 2.0]	−8.0 [−16, −1.0]	−10. [−16, 0.0]	−8.0 [−16, 4.0]
Sore throat	−4.0 [−5.0, −2.0]	−8.0 [−11, −6.0]	0.0 [−5.0, 4.0]	−4.0 [−6.0, −1.0]	−2.0 [−5.0, 2.0]	−2.0 [−4.0, 1.0]	−1.0 [−3.0, 2.0]	−4.0 [−12, 17]

**Table 3.** Median and 90% prediction interval of the maximum cross-correlation between the growth rate of Google Trends and WCIS age stratifications

Maximum growth rate cross-correlation								
Symptom	National	3 to 17 years	18 to 34 years	35 to 44 years	45 to 54 years	55 to 64 years	65 to 74 years	75 years and over
Cough	0.51 [0.41, 0.62]	0.51 [0.36, 0.65]	0.51 [0.35, 0.63]	0.51 [0.38, 0.64]	0.42 [0.34, 0.52]	0.43 [0.34, 0.52]	0.45 [0.35, 0.55]	0.43 [0.31, 0.56]
Fever	0.66 [0.49, 0.79]	0.84 [0.66, 0.94]	0.43 [0.20, 0.70]	0.63 [0.43, 0.79]	0.68 [0.48, 0.81]	0.59 [0.41, 0.73]	0.49 [0.27, 0.69]	0.24 [0.14, 0.36]
Loss of smell	0.67 [0.46, 0.83]	0.58 [0.35, 0.77]	0.48 [0.29, 0.68]	0.73 [0.57, 0.85]	0.73 [0.51, 0.89]	0.69 [0.52, 0.84]	0.50 [0.35, 0.65]	0.42 [0.31, 0.52]
Shortness of breath	0.25 [0.085, 0.44]	0.098 [−0.0073, 0.22]	0.22 [0.040, 0.44]	0.28 [0.088, 0.48]	0.22 [0.068, 0.42]	0.26 [0.098, 0.47]	0.22 [0.066, 0.42]	0.25 [0.066, 0.48]
Sore throat	0.81 [0.69, 0.90]	0.72 [0.57, 0.83]	0.62 [0.40, 0.81]	0.85 [0.73, 0.94]	0.72 [0.53, 0.87]	0.79 [0.61, 0.90]	0.80 [0.67, 0.90]	0.52 [0.29, 0.75]

In addition to the lag at which the cross-correlation is at a maximum, we also consider the value of the cross-correlation at that lag, i.e. how much the signals agree when shifted by that specific lag (Table 3). The national maximum growth rate cross-correlation varied across symptoms, with the highest value observed for sore throat (0.81 [0.69, 0.90]) and the lowest for shortness of breath (0.25 [0.085, 0.44]). The age group with the highest maximum growth rate cross-correlation also varied across symptoms. For cough, the highest maximum cross-correlation was observed for the 3 to 17 and 18 to 34 years age groups (0.51 [0.36, 0.65] and 0.51 [0.35, 0.63], respectively). Similarly, for fever, the highest cross-correlation was obtained for the 3 to 17 years age group (0.84 [0.66, 0.94]). In contrast, for sore throat, the highest maximum growth rate cross-correlation was found in the 35 to 44 age group (0.85 [0.73, 0.94]) and for loss of smell in the 35 to 44 and 45 to 54 age groups (0.73 [0.57, 0.85] and 0.73 [0.51, 0.89], respectively). For shortness of breath, the growth rate cross-correlations remained low across age groups.

We also considered the incidence cross-correlation and found similar results (see Supplementary Figure 4 and Supplementary Tables 1 and 2). The median of the lags maximizing the incidence cross-correlation was negative across all symptoms and age groups, again aligning with Google Trends lagging behind WCIS incidence. The maximum incidence cross-correlation was generally high, except for shortness of breath, and typically the highest cross-correlation was seen in the oldest age groups, except for fever, for which cross-correlation was highest in the 3 to 17 years age group.

## Discussion

In recent years, the integration of search data into epidemiological research and surveillance has garnered increasing attention. Google Trends has emerged as a tool for monitoring infectious diseases [17] and as an indicator for predictive modelling [18, 19]. This study builds upon this body of work by demonstrating that the trends in search data can reflect patterns in community symptom incidence, as estimated from the WCIS.

A key benefit of Google Trends data is its public availability, easily acquired by both organizations and individuals. Our approach aligns with this principle by using only readily available tools, rather than bespoke or highly specialized models. This not only makes reproducing our results more straightforward but also allows for our described methodology to be readily adapted by individuals outside of academic or epidemiological disciplines.

Our findings show alignment between Google Trends data and symptom incidence, particularly in terms of the timing, shape, and number of peaks. Additionally, the growth rates were comparable, indicating that even though the data were on different scales, the change in incidence direction matched. Whilst previous studies have not compared Google Trends to community symptom incidence, our findings are consistent with earlier research showing that Google Trends aligns with surveillance data influenced by healthcare-seeking behaviour [19]. This study was limited to national Google Trends, but a more spatially granular analysis could show different agreement between signals [20].

We found variations between symptoms in the timing of when Google Trends and WCIS incidence peaked, which could be explained by variation in the respiratory pathogens in circulation. For example, loss of smell only had a single peak, which occurred in late December 2023, aligning with the observed peak in COVID-19 case reports [21]. In contrast, fever had two relatively large peaks with the first occurring similarly in late December and the second occurring in late January 2024, aligning with the peak in influenza case reports [21]. This highlights a further use for the surveillance of community symptom incidence to indicate the pathogens primarily in circulation without the need for testing.

The alignment in the peaks in symptom incidence was more evident for younger age groups, although less so in the 18 to 34 years age group. Similarly, the maximum growth rate cross-correlation was generally higher for younger age groups, although the extent to which this was the case varied across symptoms. Similarly, Domnich et al. [22] found that incorporating Google Trends data improved predictions of influenza-like illness morbidity in the 0–4 and 25–44 year age groups but led to inaccuracies for the 15–24 and 65+ age groups. This reflects previous findings that those who are 65 and over were the least likely to use the Internet for health information, followed by individuals in the 18 to 24 years age group [23]. Further work is needed to develop robust estimates for the age distribution of individuals who use Google search for health-related queries, which could then be used to refine national symptom incidence estimates through poststratification.

Overall, we found that Google Trends tended to lag behind WCIS incidence, with median national estimates ranging between a lag of 3 and 12 days, depending on the symptom. Specifically, the peaks in symptom incidence occurred later for Google Trends, and the maximum growth rate cross-correlation was observed when Google Trends was shifted backwards in time. In addition, the lag was often largest in the 3 to 17 years age group, which was found to correlate well with Google Trends, indicating that this lag is likely not due to differences in the age distributions of each source. The lag could be attributed to the fact that individuals may not search for their symptoms online until after they have experienced them for a few days, or until they become sufficiently severe. This aligns with limitations observed in analyses of Google Flu Trends, which predicted the incidence peak to be up to 3 weeks later than the observed peak [24, 25].

However, for some symptoms, the lag between WCIS incidence and Google Trends was quite long, potentially too long to be fully explained by a delay between symptom onset and searching. For example, in the case of loss of smell, on average shifting Google back in time by 10 days maximized the cross-correlation of the growth rate. Furthermore, previous studies found that peaks in Google Trends preceded peaks in confirmed cases [19, 26]. The potential delay between symptom onset and searching online has not been explored in the literature.

Another potential explanation for the lag between Google Trends and WCIS incidence is that our estimates for the symptom durations from WCIS data were too long. Since we shifted our estimate back in time according to the estimated symptom duration as part of our transformation from prevalence to incidence, overestimating the duration would cause WCIS incidence to lag Google Trends. For example, we estimated that the mean duration of fever would be at least 9 days across all age groups; however, generally, the duration of fever is estimated to be between 1 and 5 days [27, 28].

Several factors may have contributed to a potential overestimation of the symptom durations. Firstly, our symptom duration estimates were based on self-reported symptom onset dates, which ranged from several days to several hundred days in the past, indicating that both chronic and acute symptoms were represented in the data. Furthermore, due to the construction of the survey, only a single symptom onset date could be given, even if an individual experienced multiple symptoms. The symptom onset date was then the earliest date at which one of the symptoms started. Finally, the individual may have stopped experiencing the symptom up to 7 days before they submitted the survey, again leading to exaggerated symptom durations.

In conclusion, this study demonstrates that Google Trends can serve as a valuable tool for monitoring community symptom incidence, aligning well with patterns observed in the WCIS data. Whilst our findings indicate some strong relationships between search Trends and symptom incidence, the observed lag between Google Trends and WCIS indicates the need for further study into the timing of health-related search behaviour. Future studies with the explicit goal of monitoring symptom incidence, ideally through more frequent testing and symptom reporting, and individual onset dates for each symptom, will be essential to validate these findings and enhance our understanding of the accuracy of Google Trends as a proxy for community symptom incidence.

## Methods

### Study info

The WCIS was jointly run by the UK Health Security Agency and the Office for National Statistics. A cohort of randomly sampled households was recruited and asked to complete questionnaires monthly from 13 November 2023 to 7 March 2024. Participants were cohorted into weekly waves, with staggered start dates for each survey window. There were 111,752 participants, with 386,343 submissions completed during the study. Not all participants responded in every wave of the survey (Supplementary Table 3). The cohort consisted of adults aged three and over in England, living in residential settings. Participants were representative of all regions, though skewed towards older and wealthier individuals (Supplementary Table 4).

Along with demographic information, participants were asked questions about their symptomatic status, such as ‘Have you had any of the following symptoms in the last 7 days?’ and ‘Which of these symptoms are new or worse than usual for you?’ The count and percentage of the total symptomatic statuses reported by respondents are given in Supplementary Table 5. Multiple modelling steps are required to produce a representative symptom incidence from this data.

To compare symptom incidence, we also use the symptom search dataset (SSD) from Google Research [11]. The data represent a daily national-level relative search volume for over 400 symptoms, originally set up to monitor SARS-CoV-2. The SSD represents a signal of individuals in aggregate searching for information about symptoms, relative to total search volume.

In this work, we explore the following symptoms relating to winter respiratory illnesses: ‘cough’, ‘fever’, ‘loss of smell’, ‘shortness of breath’, and ‘sore throat’.

### Google incidence

Throughout, we assume that a Google search for a symptom is a proxy for symptom onset as an individual seeks to learn more about their new symptom. Whilst true incidence itself can vary smoothly over time at a population level, observations of symptom incidence are influenced by behaviour changes and reporting effects. Namely, healthcare-seeking behaviour varies by day of the week and can be impacted by holiday periods. We therefore fit a generalized additive model (GAM) to adjust for these known biases in the reporting of syndromic surveillance Google SSD.

The relative search volume (RV) at time t, V(t), is continuous and bounded by (0,100). The rescaled search volume, denoted *S*(*t*) = *V*(*t*)/100 ∈ (0,1), is assumed to follow a Beta distribution

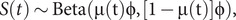




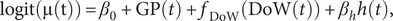

where,

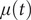
 is the 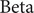 mean on the logit scale; 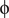 is a single precision parameter estimated by restricted maximum likelihood (REML).

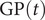
 is a Gaussian-process spline that imposes a squared-exponential correlation structure decaying with the squared time difference between days.

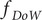
 is a cyclic cubic spline of day-of-week.

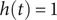
 on UK public holidays, 0 otherwise.

The terms 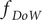 and 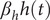 are marginalized out, providing a smooth estimate of RV over time. Prediction samples are generated from the model coefficients using a Metropolis-Hastings approach, giving 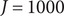 samples of the symptom incidence, 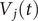 , from which the median and 90% prediction intervals are generated. The model is fit using *mgcv* [29] and prediction samples extracted with *gratia* [30].

### WCIS incidence method

To compare Google Trends with a reliable baseline, we require the daily incidence of new symptoms from WCIS. Note that, whilst we could straightforwardly estimate the incidence of symptoms within the study participants from their symptom onset dates, it would not be possible to directly extend this to a symptom incidence rate across the whole population. This would require knowledge of participation rates, which cannot be defined with respect to symptom onset dates. In contrast, we can more easily estimate prevalence rates because we have data on both individuals who do have the symptom and those who do not at each time point. Furthermore, the symptom onset date provided in the study is a single onset date for all symptoms, which forces the assumption of concurrent onsets of each symptom.

Therefore, incidence is not observed directly but can be reconstructed from the prediction draws of prevalence and symptom duration.

Let 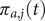 be the 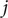 -th prevalence prediction draw for age group 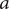 on day 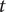 , and let 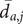 be the corresponding draw of the mean symptom duration. If we approximate every episode as lasting exactly 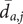 days, then prevalence and incidence are linked by

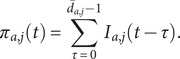



Therefore,

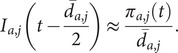

This is calculated by dividing each prevalence draw by its paired mean duration and shifting the resulting incidence curve **backwards** by 
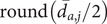
 . This produces a set of incidence draws 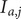 that match the daily resolution of the WCIS data and carry the full uncertainty from both prevalence and duration estimates. Further information on the derivation of incidence can be found in Supplementary Section 2.

### WCIS prevalence

We estimated the proportion of participants who were currently symptomatic by combining a multilevel-regression-and-poststratification (MRP) framework [31] with Gaussian-process smoothers previously developed for influenza-like illness [32]. Sub-groups are defined by age group 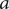 , region 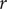 (nine English regions), and invitation window index, 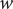 , defined to be the time between the response window start date and the survey submission date.

For each calendar day, 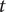 , the survey yields 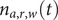 symptomatic responses out of 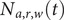 returned questionnaires. We assume the number of symptomatic responses follows a binomial distribution with 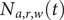 as the number of trials and prevalence 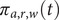 as the probability of success:

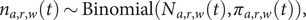




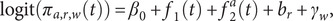

where,

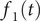
 is the national GP smoother

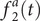
 is an age-specific GP smoother

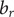
 is a Gaussian random intercept for region

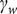
 is a categorical fixed effect for time from response window start to participant submission date.

#### Prediction sampling and marginalization

We draw 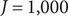 prediction samples from the model using *gratia*, which randomizes all smoothing parameters via a Metropolis–Hastings step. To obtain window-marginal prevalences, we weight each 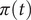 by the frequency of its window:

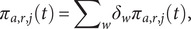

where,

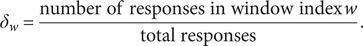



#### Poststratification

Census population totals 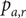 are treated as fixed. For every draw 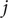




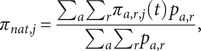




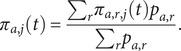



The resulting national time-series 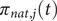 provide 1,000 prediction draws of daily national and age-specific prevalence, used for downstream incidence calculation.

### WCIS symptom duration

We modelled the distribution of symptom durations reported by WCIS participants using the R package *epidist* (v0.3.0 [33]) via Bayesian MCMC, allowing for interval censoring and right truncation [34, 35]. Let 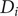 be the true duration (in days) of episode 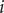 reported by a participant in age group 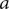 . We assume a log-normal duration model:

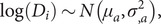

with age group specific parameters

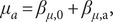




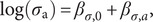

where 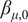 and 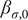 are global intercepts, and 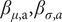 are fixed effects for each of the seven age bands. We select priors for the intercept terms with mean and standard deviation parameterization of a normal distribution,

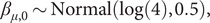




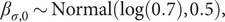

and uninformative priors for the age-specific fixed effects,

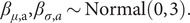



#### Censoring and truncation

- **Onset times** were interval censored to 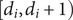 days, where 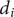 is the date that the participant reported symptom onset.
- **End times** were right-truncated to lie in 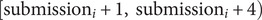 days, allowing up to 3 days of unreported recovery.
- Episodes implying durations >60 days (0.4% of records) were excluded to remove potential chronic conditions.

For each posterior draw j and age group a, we computed the mean duration

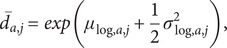



We summarize the distribution of 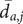 by its posterior median and 90% credible intervals.

We assumed that symptoms started within a one-day window after the symptom onset date provided by the participant and ended at most 3 days after the survey submission date. We explored the sensitivity to the choice of window length for the symptom end date in Supplementary Figure 5. We found that increasing the window length increased the estimate of the mean duration across age groups, and inclusion of a short window reduced the uncertainty in our estimates.

Furthermore, individuals were surveyed multiple times throughout the study period; however, we assumed these surveys were independent due to being sufficiently far apart (around one month between responses) for individuals to have recovered from an infection event. In addition, some respondents gave symptom onset dates many months in the past, despite the survey focusing on symptoms that have recently developed (or recently become worse than usual); a substantial number gave a symptom onset date of exactly one year ago, the earliest date allowed to be inputted. To avoid these longer-term, chronic symptoms skewing our duration distributions, we excluded individuals with symptom durations longer than 60 days.

### WCIS incidence implementation

For each age group, we have 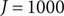 prediction draws of

- Prevalence 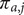 (t),
- Mean symptom duration 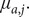

Because the duration model is fitted independently of the prevalence model, we treat the two sets of draws as independent and pair them by index 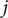 .

For every pair ( 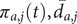 ), we approximate incidence as

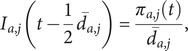

 and then shift the series **backwards** 
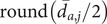
 days.

The back-shift differs across draws, so we restrict each 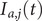 to the common window:

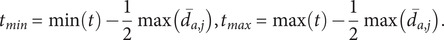



Age-specific incidence is summarized at each 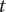 by the median and 90% prediction interval of the aligned draws, giving 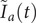 .

National incidence for draw 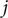 is

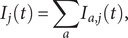

and is summarized to yield 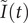 with its 90% prediction intervals.

### Growth rates

In addition to comparing the Google symptom incidence, 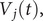 against WCIS symptom incidence, 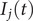 , we also compared the growth rates for each sample. Since we have discrete values for the incidence at each time step, 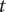 , we use the following approximation for the WCIS growth rate

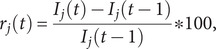

and similarly for Google symptom incidence.

The growth rates across samples were summarized to give median estimates and 90% prediction intervals.

### Cross-correlation function (with uncertainty)

Each Google incidence sample, 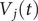 , is paired with a WCIS incidence sample, 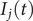 , for each symptom, with their means defined as 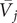 and 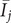 , respectively. The cross-correlation was calculated for each pair of samples, for lags, 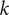 , between −17 and +17 days,

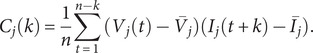



Here, a positive lag compares WCIS incidence *k* days in the future against Google Trends. The cross-correlations across samples were then summarized to give a median estimate, 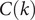 , and a 90% prediction interval for each lag considered.

Similarly, we calculated cross-correlations for Google and WCIS incidence growth rates, again summarizing to give a median estimate and 90% prediction intervals for each lag considered.

## Supporting information

10.1017/S0950268825100794.sm001 Asplin et al. supplementary material Asplin et al. supplementary material

## Data Availability

UKHSA operates a robust governance process for applying to access protected data that considers: the benefits and risks of how the data will be used compliance with policy, regulatory and ethical obligations data minimization how the confidentiality, integrity, and availability will be maintained retention, archival, and disposal requirements best practice for protecting data, including the application of ‘privacy by design and by default’, emerging privacy conserving technologies and contractual controls. Access to protected data is always strictly controlled using legally binding data sharing contracts. UKHSA welcomes data applications from organizations looking to use protected data for public health purposes. To request an application pack or discuss a request for UKHSA data you would like to submit, contact DataAccess@ukhsa.gov.uk.

## Supplementary material

The supplementary material for this article can be found at http://doi.org/10.1017/S0950268825100794.

## Code availability

The analytical code developed for this project is available: https://github.com/jonathonmellor/symptom-incidence.

## Patient and public participation

Patient and public participation was not deemed necessary for this research beyond the routine engagement of survey participants.
