# Supplementary material for: Evaluating Google Trends as a proxy for symptom incidence: insights from the winter COVID-19 infection study in England 2023/24
Source: Epidemiol Infect. 2025 Nov 28;153:e136. doi: 10.1017/S0950268825100794 (PMC12722558; doi:10.1017/S0950268825100794)
Supplement: Asplin et al. supplementary material [file S0950268825100794sup001.docx]

### Supplementary Section 1: Additional Figures


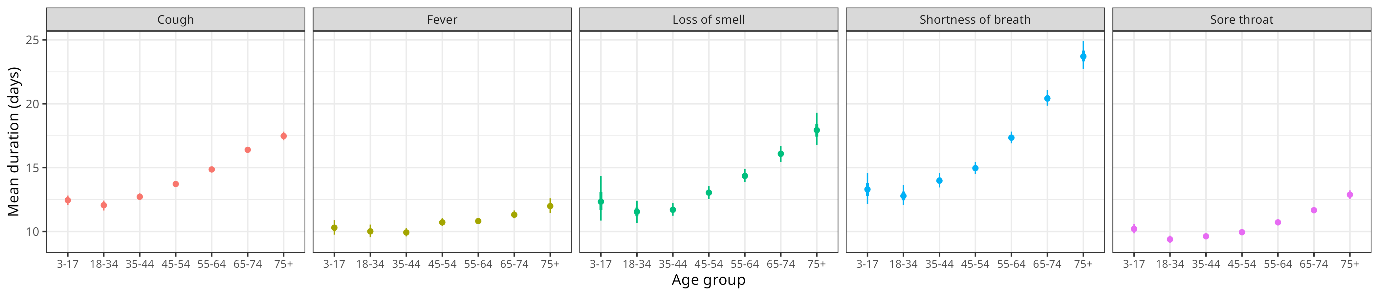

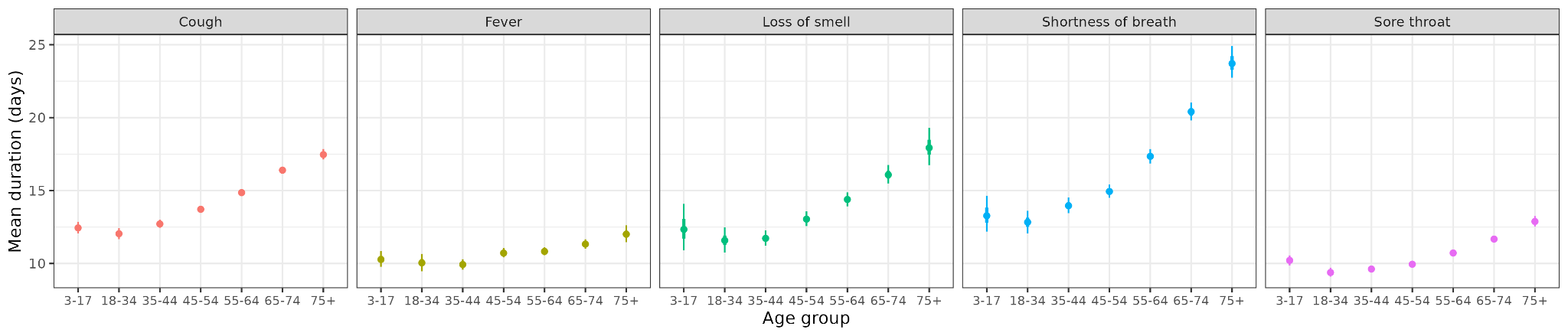


**Supplementary Figure 1.** Mean and 90% credible interval of symptom duration by age group and symptom from WCIS survey data.


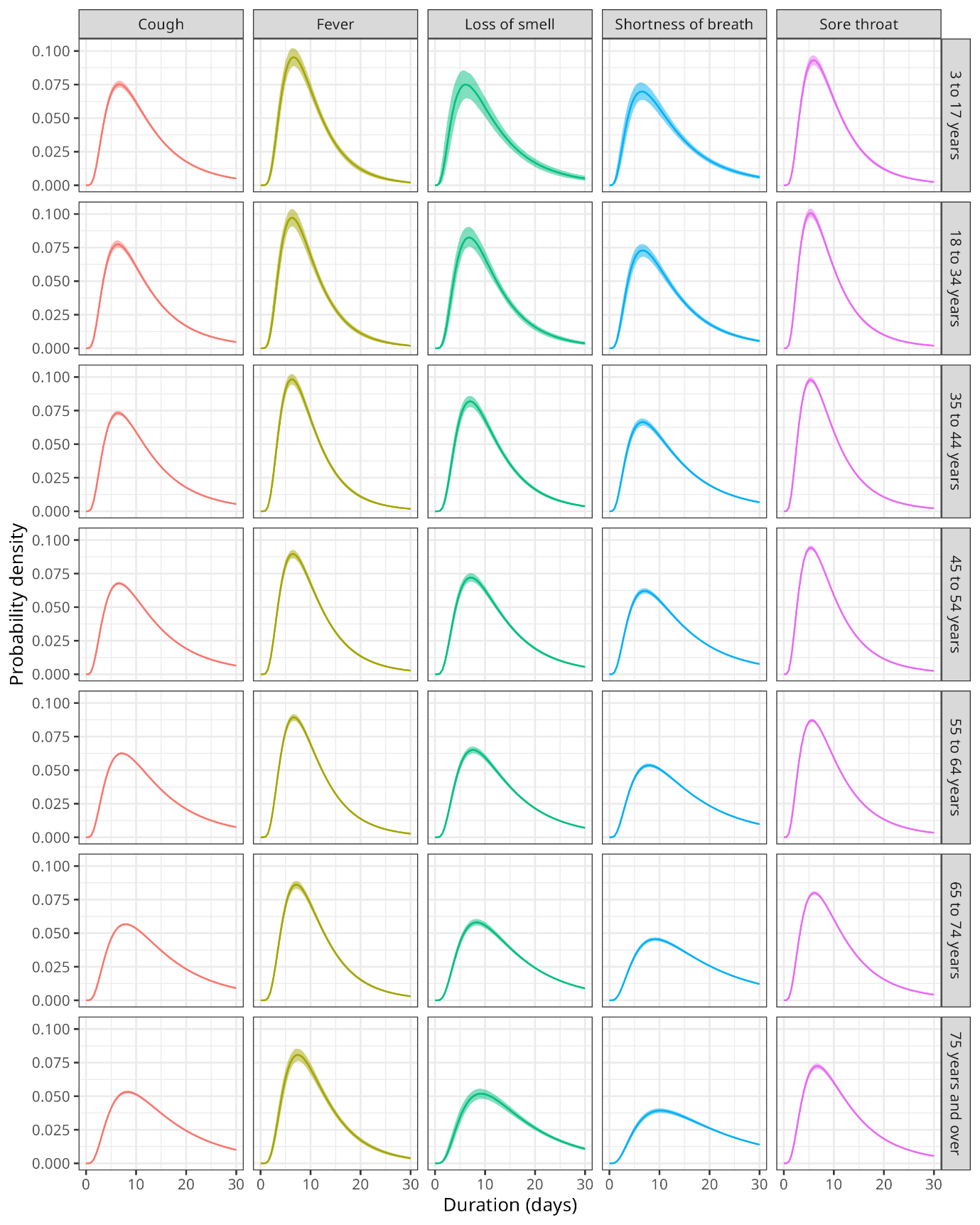


**Supplementary Figure 2.** Probability mass distribution of symptom duration by symptom and age group.


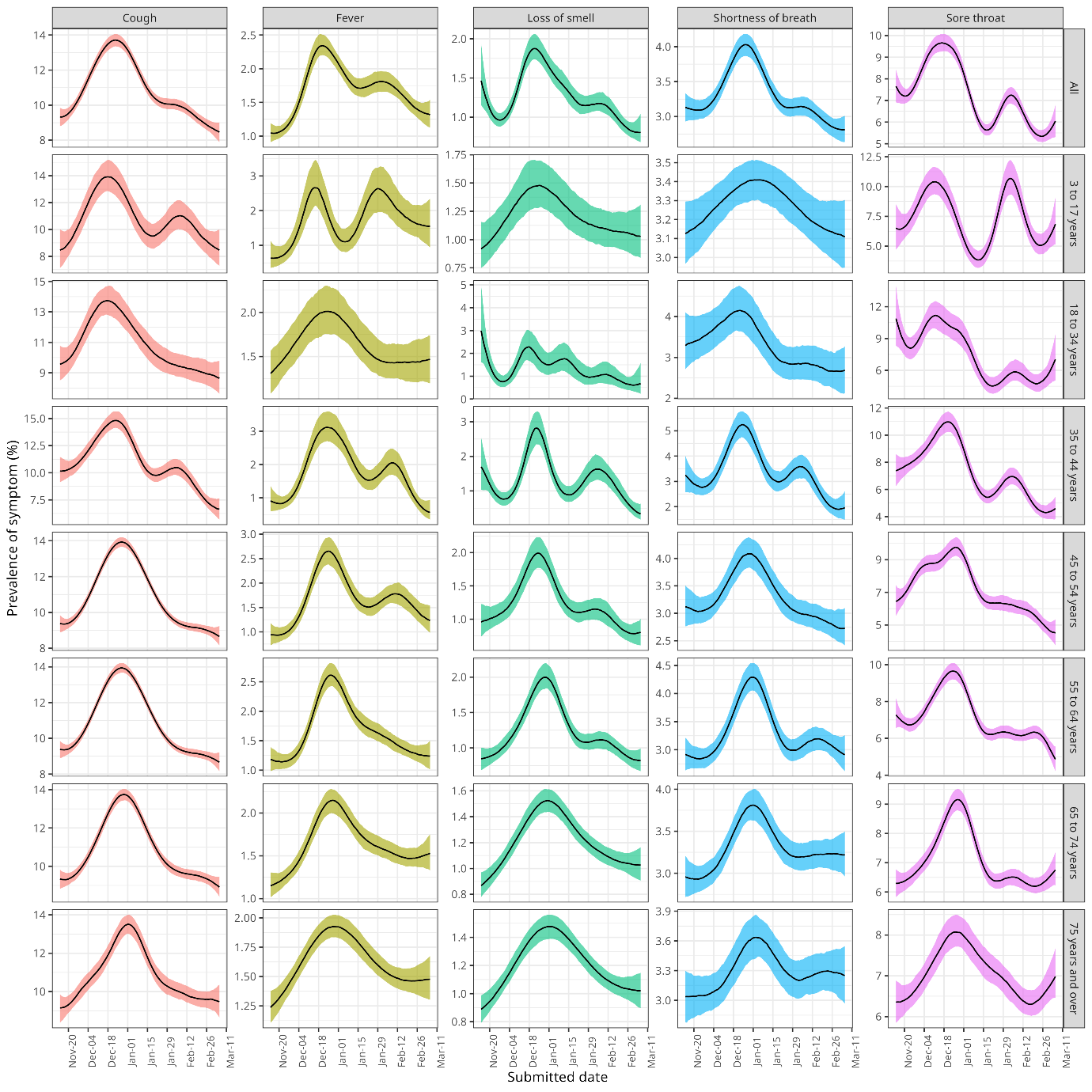


**Supplementary Figure 3.** WCIS symptom prevalence over time by submission date stratified by symptom and age group.


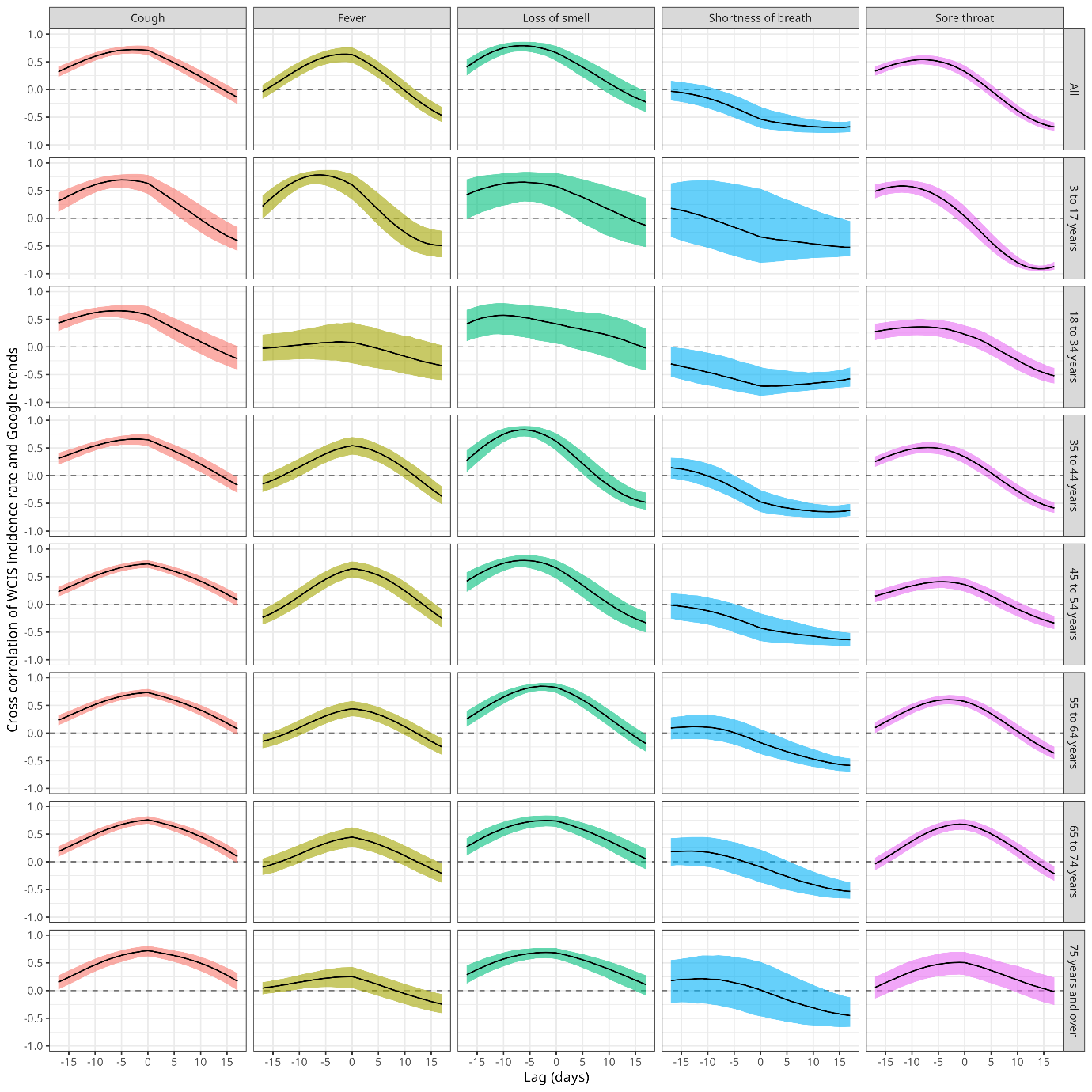


**Supplementary Figure 4.** cross-correlation at lags -17 to 17 between the Google Trends symptoms and WCIS incidence by age group stratification.

| **Lag with maximum incidence cross-correlation** | | | | | | | | |
| --- | --- | --- | --- | --- | --- | --- | --- | --- |
| **Symptom** | **All** | **3 to 17 years** | **18 to 34 years** | **35 to 44 years** | **45 to 54 years** | **55 to 64 years** | **65 to 74 years** | **75 years and over** |
| Cough | 0.51 [0.41, 0.62] | 0.51 [0.36, 0.65] | 0.51 [0.35, 0.63] | 0.51 [0.38, 0.64] | 0.42 [0.34, 0.52] | 0.43 [0.34, 0.52] | 0.45 [0.35, 0.55] | 0.43 [0.31, 0.56] |
| Fever | 0.66 [0.49, 0.79] | 0.84 [0.66, 0.94] | 0.43 [0.20, 0.70] | 0.63 [0.43, 0.79] | 0.68 [0.48, 0.81] | 0.59 [0.41, 0.73] | 0.49 [0.27, 0.69] | 0.24 [0.14, 0.36] |
| Loss of smell | 0.67 [0.46, 0.83] | 0.58 [0.35, 0.77] | 0.48 [0.29, 0.68] | 0.73 [0.57, 0.85] | 0.73 [0.51, 0.89] | 0.69 [0.52, 0.84] | 0.50 [0.35, 0.65] | 0.42 [0.31, 0.52] |
| Shortness of breath | 0.25 [0.085, 0.44] | 0.098 [-0.0073, 0.22] | 0.22 [0.040, 0.44] | 0.28 [0.088, 0.48] | 0.22 [0.068, 0.42] | 0.26 [0.098, 0.47] | 0.22 [0.066, 0.42] | 0.25 [0.066, 0.48] |
| Sore throat | 0.81 [0.69, 0.90] | 0.72 [0.57, 0.83] | 0.62 [0.40, 0.81] | 0.85 [0.73, 0.94] | 0.72 [0.53, 0.87] | 0.79 [0.61, 0.90] | 0.80 [0.67, 0.90] | 0.52 [0.29, 0.75] |

**Supplementary Table 1.** Median and 90% prediction interval of the lag with the maximum cross-correlation between the incidence of Google trends and WCIS age stratifications.

| **Maximum incidence cross-correlation** | | | | | | | | |
| --- | --- | --- | --- | --- | --- | --- | --- | --- |
| **Symptom** | **All** | **3 to 17 years** | **18 to 34 years** | **35 to 44 years** | **45 to 54 years** | **55 to 64 years** | **65 to 74 years** | **75 years and over** |
| Cough | 0.73 [0.65, 0.80] | 0.71 [0.57, 0.81] | 0.67 [0.56, 0.77] | 0.66 [0.57, 0.75] | 0.73 [0.66, 0.80] | 0.73 [0.66, 0.80] | 0.76 [0.69, 0.82] | 0.72 [0.62, 0.81] |
| Fever | 0.65 [0.51, 0.77] | 0.80 [0.64, 0.88] | 0.16 [-0.045, 0.49] | 0.55 [0.39, 0.70] | 0.65 [0.49, 0.78] | 0.44 [0.31, 0.58] | 0.45 [0.27, 0.62] | 0.26 [0.072, 0.43] |
| Loss of smell | 0.80 [0.71, 0.87] | 0.72 [0.45, 0.87] | 0.65 [0.44, 0.82] | 0.84 [0.73, 0.91] | 0.81 [0.69, 0.90] | 0.85 [0.77, 0.91] | 0.76 [0.65, 0.85] | 0.70 [0.59, 0.78] |
| Shortness of breath | -0.030 [-0.20, 0.16] | 0.18 [-0.32, 0.70] | -0.30 [-0.48, 0.017] | 0.15 [-0.051, 0.33] | -0.0070 [-0.25, 0.20] | 0.13 [-0.089, 0.34] | 0.21 [-0.038, 0.46] | 0.26 [-0.15, 0.67] |
| Sore throat | 0.54 [0.46, 0.62] | 0.60 [0.47, 0.69] | 0.39 [0.24, 0.52] | 0.51 [0.41, 0.61] | 0.41 [0.30, 0.51] | 0.61 [0.53, 0.69] | 0.68 [0.58, 0.77] | 0.53 [0.31, 0.71] |

**Supplementary Table 2.** Median and 90% credible interval of the maximum cross-correlation between the incidence of Google trends and WCIS age stratifications.


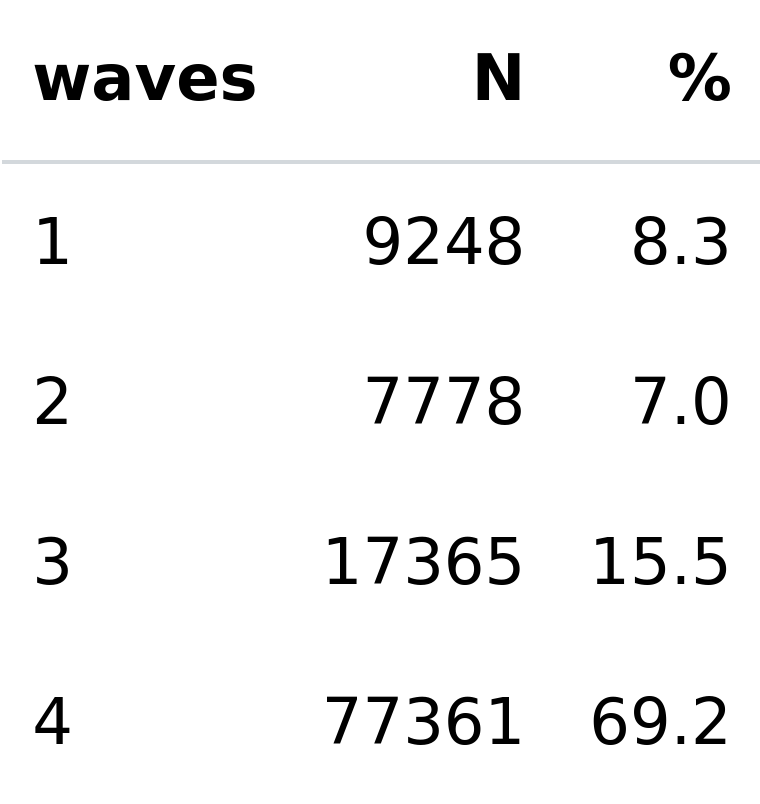


**Supplementary Table 3.** The count and percentage of the cohort by total waves responded to during the study.


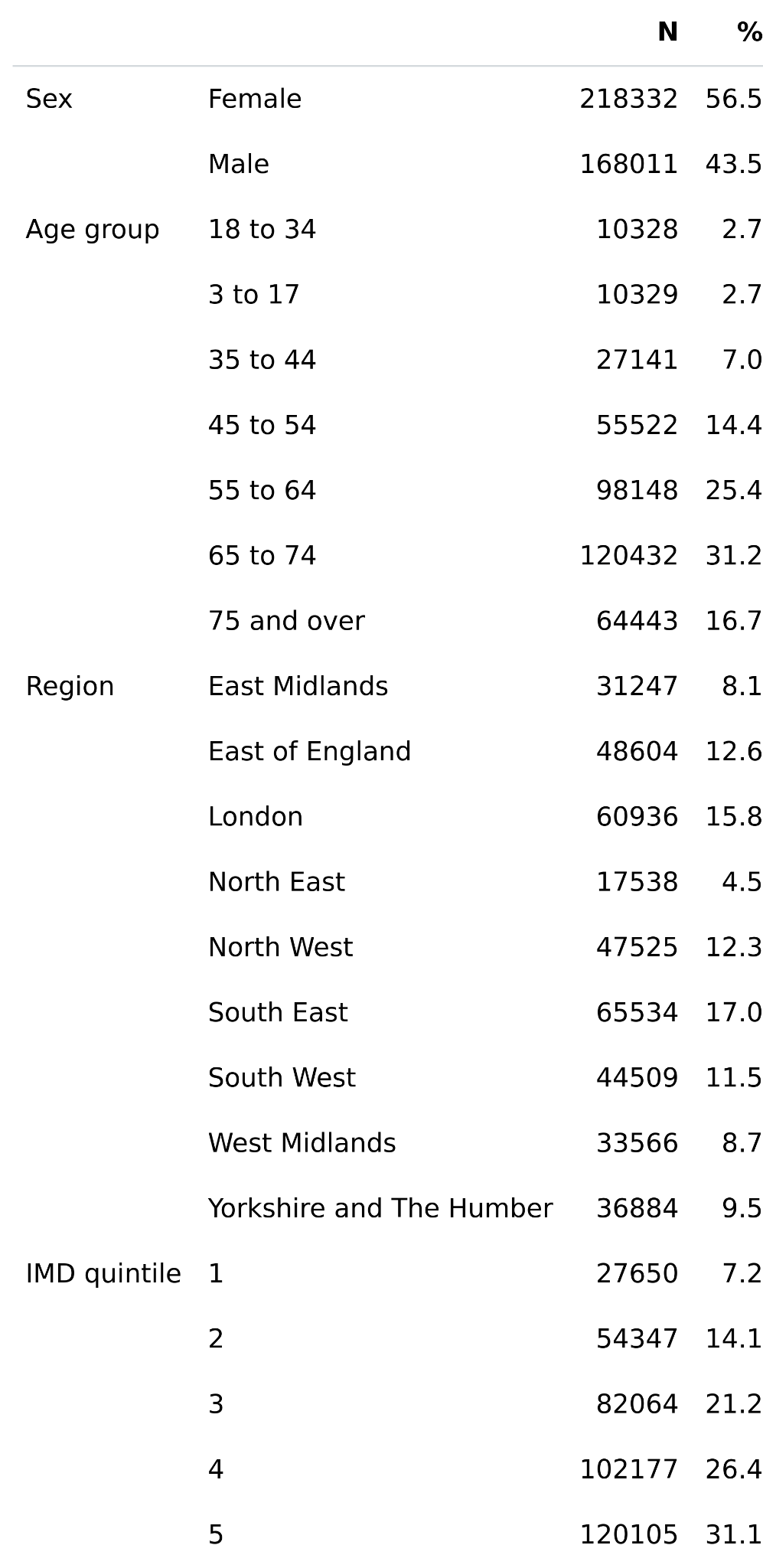


**Supplementary Table 4.** Count and percentage of the total responses from the cohort by sex, age group, region and IMD quintile in the WCIS study.


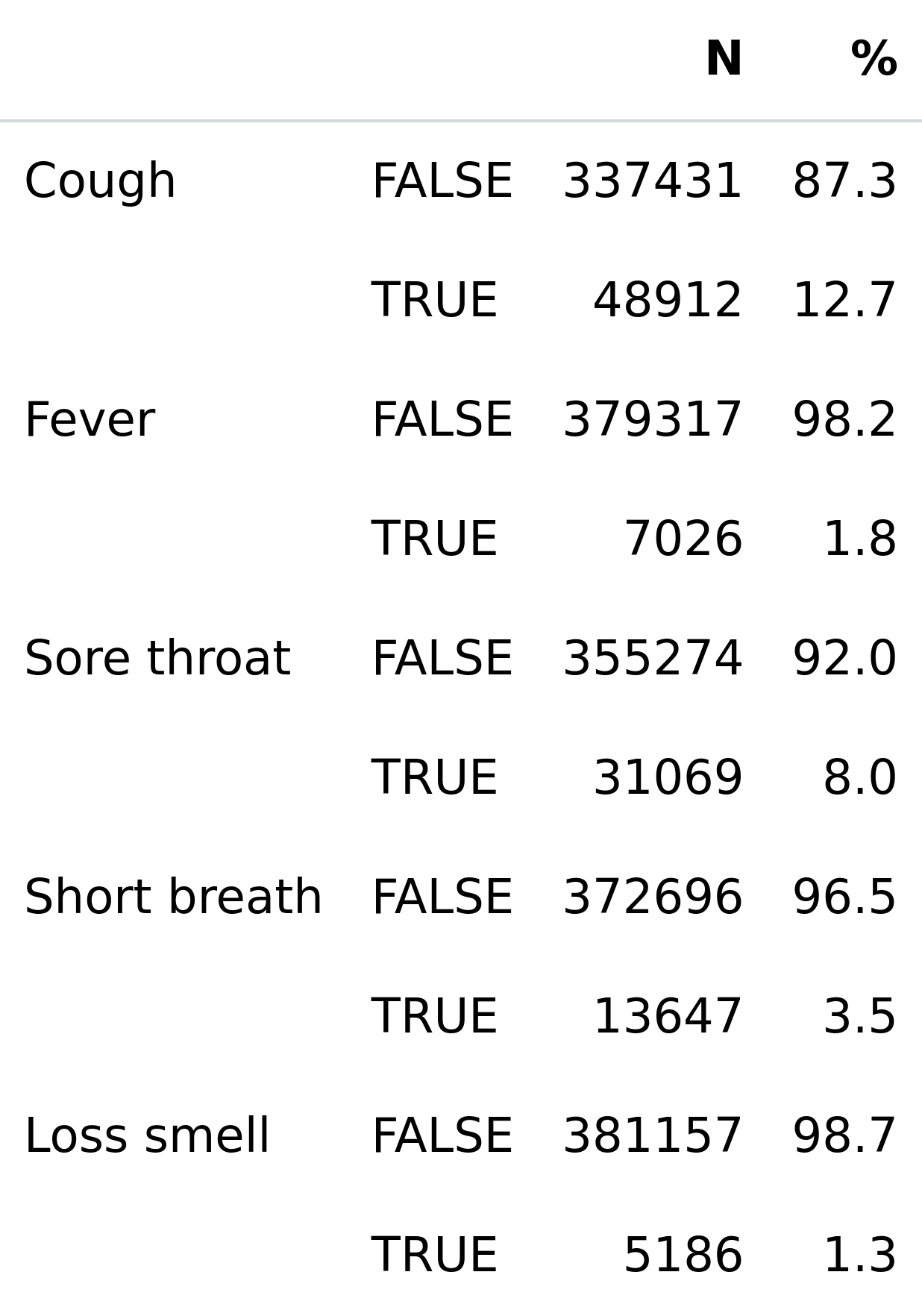


**Supplementary Table 5.** Count and percentage of the total symptomatic statuses reported by respondents in the WCIS study.


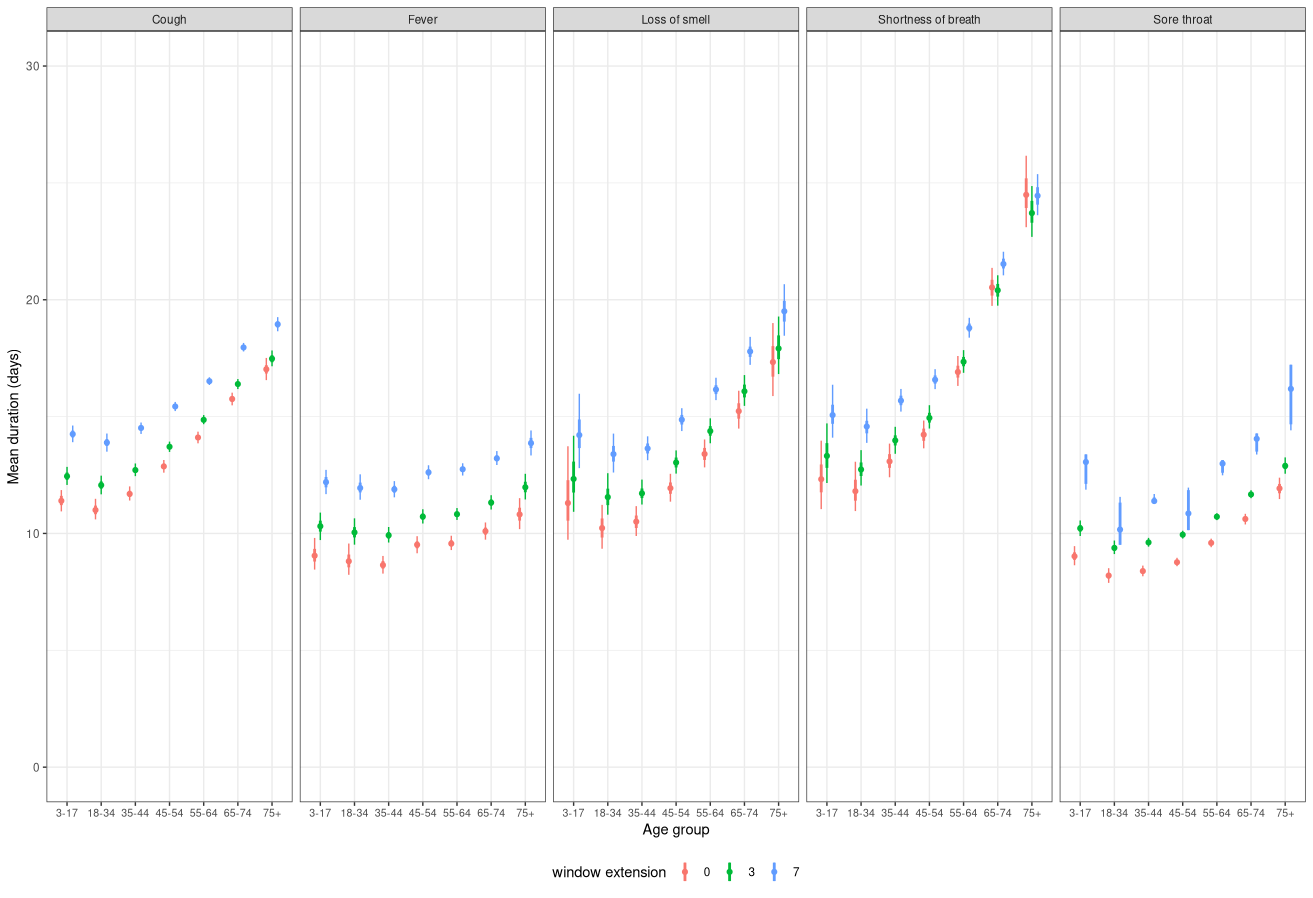


**Supplementary Figure 5.** Sensitivity of the duration of symptomatic status by second event window length, stratified by symptom and age group.

### Supplementary Section 2: Incidence Derivation

To understand Google Trends in the context of a reliable baseline, we need to compare symptom incidence (i.e. the daily rates of new symptom onsets). WCIS symptom incidence can be obtained via a backwards approximation from estimated prevalence. Firstly, an epidemic time series $F\left( t \right)$ can be calculated as a convolution of some function $f\left( \tau\right)$ and the time series of incidence itself $I\left( t \right)$

$F\left( t \right)= \int_{0}^{\infty} I\left( t-\tau\right)f\left( \tau\right) d\tau$.

In our context, the prevalence $\pi\left( t \right)$ can be obtained from incidence using the probability of having symptoms $\tau$ days after onset,

$\pi\left( t \right)= \int_{0}^{\infty} I\left( t-\tau\right)f_{prev}\left( \tau\right) d\tau$.

We assume all individuals have symptoms for exactly $\mu$days, where $\mu$ is the mean symptom duration. Therefore, $f\left( \tau\right)= 1$ when $\tau\in\left[ 0, \mu\right]$ and $f\left( \tau\right)= 0$ otherwise. This gives

$\pi\left( t \right)= \int_{0}^{\mu} I\left( t-\tau\right) d\tau\approx\mu I\left( t -\frac{\mu}{2} \right)$.

Meaning we can obtain incidence as,

$I\left( t -\frac{\mu}{2} \right)= \frac{\pi\left( t \right)}{\mu}$,

motivating us to estimate symptom prevalence over time, $\pi\left( t \right)$ and average symptom duration, $\mu$.

### Supplementary Section 3: Deconvolution Sensitivity

The back-shift approach is an approximation assuming that the duration of symptoms is a fixed value, and that incidence does not rapidly change, which are not strictly correct. Using an alternative approximation method, which relaxes the fixed duration assumption, we can improve our understanding of the appropriateness of the back-shift method.

The Richardson-Lucy (RL) deconvolution method used by Goldstein et al [1] takes an input timeseries, a time delay distribution and iteratively fits a delay distribution based shifted curve, using the *fastbeta* R package [2]. Dividing this by the expected duration of symptoms gives us the expected incidence curve.

The RL method is not well match at the tails of the time series, we therefore right truncate to the backward shifted end date and avoid drawing conclusions from the first few days of the deconvolution approach.

In Supplementary Figure 6, both the backward shifted and deconvolution methods are shown together for symptoms and age groups. Nationally, symptoms where the overall magnitude change is small in the time series, such as fever and loss of smell, the two methods match well. In all cases the shapes of the curves match, though with varying magnitudes in some instances. Generally, the deconvolution approach has more pronounced peaks and troughs. This may be partly due to where the “slow moving” assumption of the backward shift approximation fails, or an effect of the iterative algorithm converging at different points based on the scale of the time series. A range of tolerance threshold values were explored for the RL algorithm.

Supplementary Figure 7 shows the relative difference between the two approaches, with a clear divergence at the start of the time series due to boundary effects, and oscillating difference corresponding the peaks and troughs of the symptom waves.


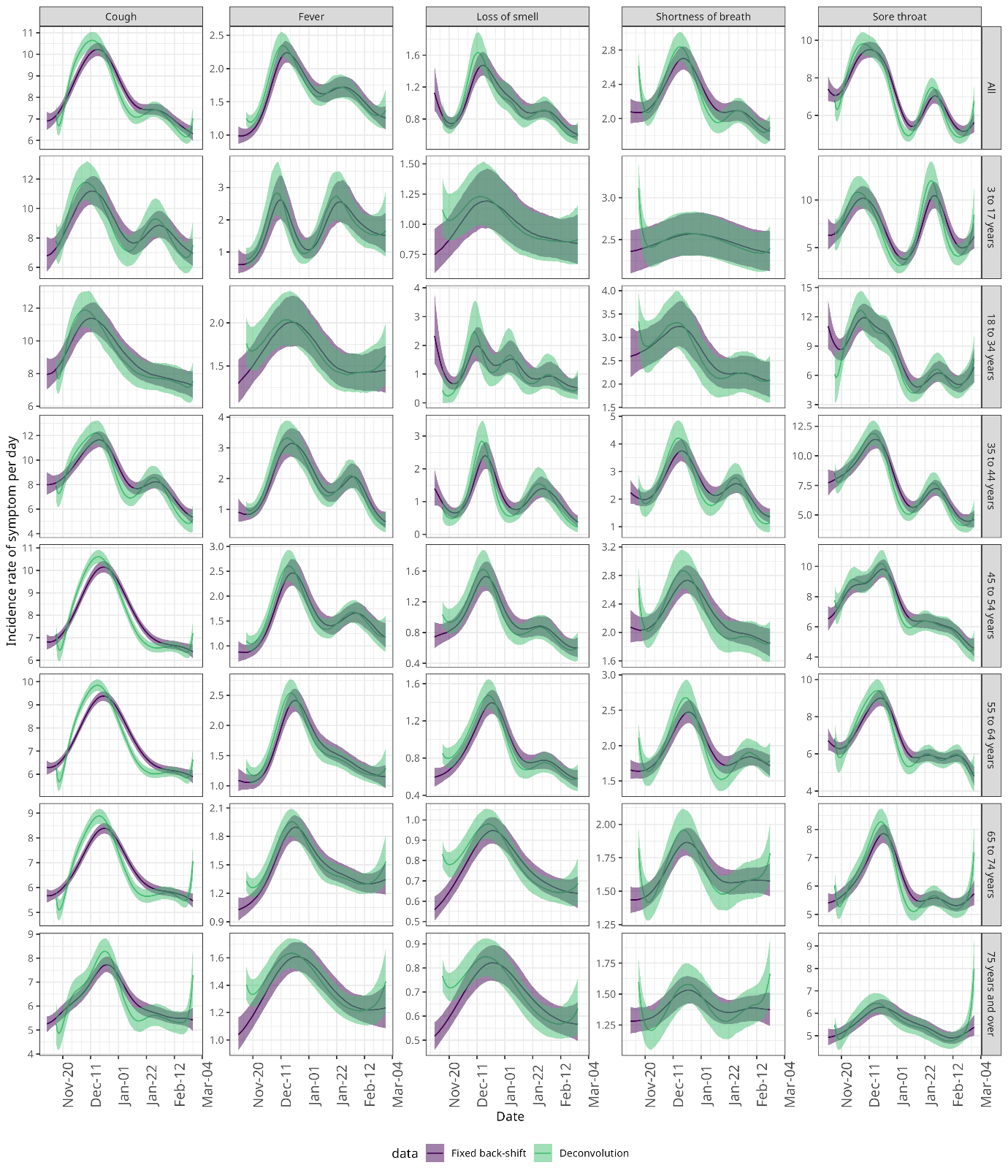


**Supplementary Figure 6.** Comparison of backward shift versus deconvolution method for prevalence to incidence calculation.


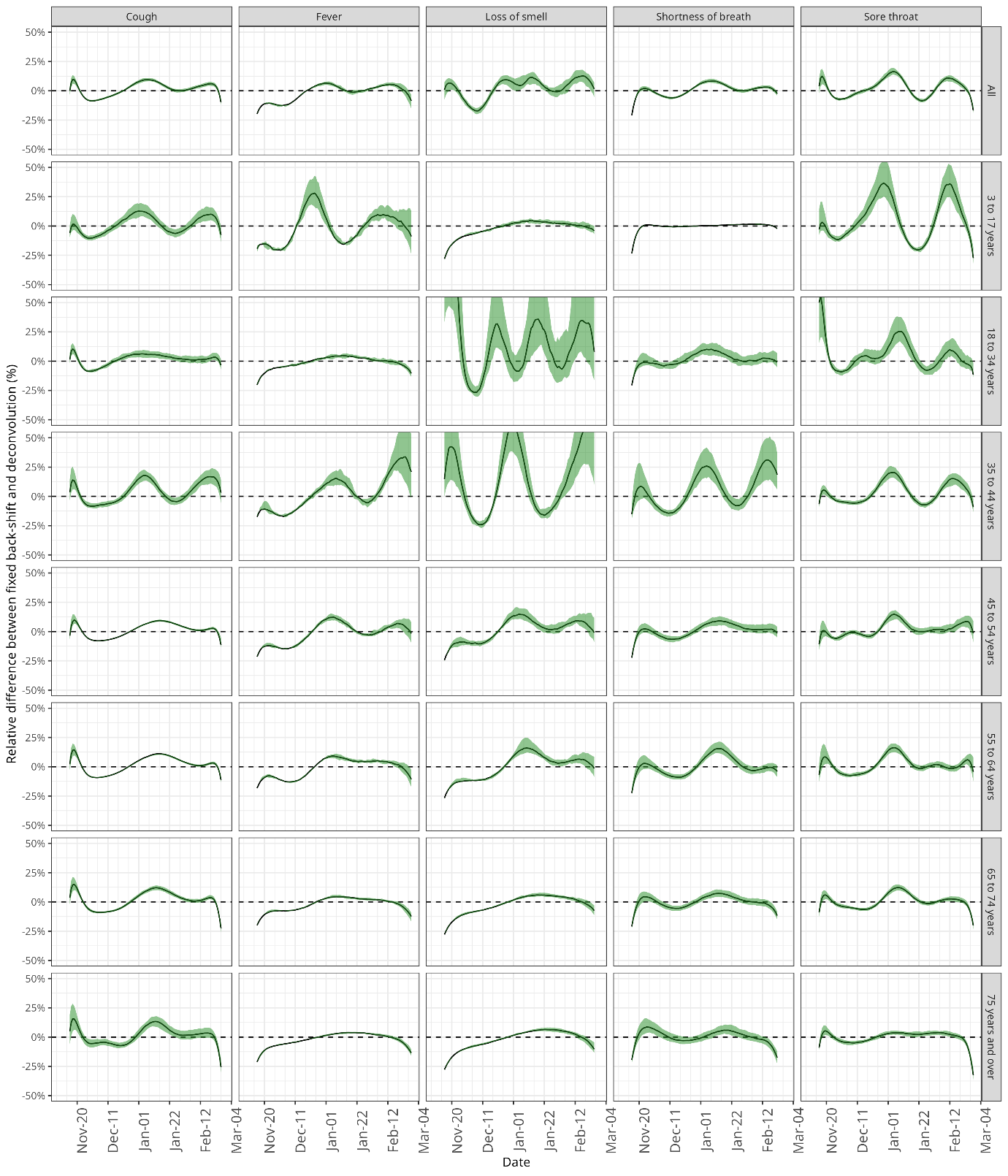


**Supplementary Figure 7.** Relative difference between the deconvolution and fixed back-shift method showing how well they correspond. The median estimates are compared, as are the 90% intervals.

# References

| [1] | E. Goldstein, J. Dushoff, J. Ma, J. B. Plotkin, D. J. Earn and M. Lipsitch, “Reconstructing influenza incidence by deconvolution of daily mortality time series,” *Proceedings of the National Academy of Sciences,* vol. 106, no. 51, pp. 21825-21829, 2009. |
| --- | --- |
| [2] | M. Jagan, “Fast Approximation of Time-Varying Infectious Disease,” 22 July 2025. [Online]. Available: https://cran.r-project.org/web/packages/fastbeta/fastbeta.pdf. [Accessed 2025]. |
